# Supplementary material for: Bacterial Lipopolysaccharide Induces PD-L1 Expression and an Invasive Phenotype of Oral Squamous Cell Carcinoma Cells
Source: Cancers (Basel). 2024 Jan 13;16(2):343. doi: 10.3390/cancers16020343 (PMC10813992; doi:10.3390/cancers16020343)
Supplement: Supplementary file 1 [file cancers-16-00343-s001.zip › cancers-2784386-supplementary.pdf]

Table1

Name	Company	Cat no.
Actin(c4) mouse mAb	SANTACRUZ	sc-47778
TLR4 Polyclonal antibody rabbit mAb	proteintech	19811-1-AP
E-cadherin(24E10) rabbit mAb	Cell signaling Technology	3195
N-cadherin(13A9) rabbit mAb	SANTACRUZ	sc-7939
PD-L1(28-8) rabbit mAb	Abcam	ab213524
LAMB3(OTI2D11) mouse mAb	OriGene	TA804403
TSG101(EPR7130) rabbit mAb	Abcam	ab125011
CD63(190523-001) goat mAb	System Bioscience	EXOAB-CD63A-1

Table2

	Forward	Reverse	bp
MMP-2	GGCTCATGCCTTCGCCCCAG	ACTCCCCATCGGCGTTCCCA	122
MMP-9	TGACAGCGACAAGAAGTG	CAGTGAAGCGGTACATAGG	143
Zeb1	TCCATGCTTAAGAGCGCTAGCT	ACCGTAGTTGAGTAGGTGTATGCCA	78
Zeb2	CAAGAGGCGCAAACAAGCC	GGTTGGCAATACCGTCATCC	128
Snail1	GGCGCACCTGCTCGGGGAGTG	GCCGATTCGCGCAGCA	199
Snail2	GGGGAGAAGCCTTTTTCTTG	TCCTCATGTTTGTGCAGGAG	158
GAPDH	ACCATCTTCCAGGAGCGAGA	ACCACCTGGTGCTCAGTGTA	623
